# IDEAL Phase 1 evaluation of subungual tube placement with selective nail-fold wedge resection for moderate-to-severe onychocryptosis-associated paronychia

**DOI:** 10.3389/fmed.2026.1822211

**Published:** 2026-08-12

**Authors:** Tianhao Li, Jingni Liang, Jingchun Li, Fan Yang, Fei Tian, Min Xiao, Chunguang Xie

**Affiliations:** 1 Hospital of Chengdu University of Traditional Chinese Medicine, Chengdu, China; 2 School of Clinical Medicine, Chengdu University of Traditional Chinese Medicine, Chengdu, China; 3 The First People’s Hospital of Dazhou, Dazhou, China

**Keywords:** IDEAL framework, onychocryptosis-associated paronychia, recurrence, selective nail-fold wedge resection, subungual tube placement

## Abstract

**Background:**

Onychocryptosis-associated paronychia is a common condition with frequent recurrence after conventional treatment. Minimally invasive, nail-preserving strategies remain insufficiently standardized and have not been extensively evaluated within structured clinical-development frameworks. This study reports the findings of an IDEAL Phase 1 (Idea) study investigating the technical feasibility, early safety, and preliminary clinical outcomes of subungual tube placement combined with selective nail-fold wedge resection for the treatment of moderate-to-severe onychocryptosis-associated paronychia.

**Methods:**

This prospective, single-arm, single-center case series enrolled 11 consecutive patients with moderate-to-severe onychocryptosis-associated paronychia. According to predefined criteria based on the severity of nail-fold hypertrophy and fibrosis, three patients underwent subungual tube placement combined with selective nail-fold wedge resection, whereas eight underwent isolated subungual tube placement. Outcomes included healing time, pain intensity measured using the Visual Analog Scale (VAS), and recurrence rate.

**Results:**

All procedures were completed successfully. The mean healing time was 5.8 weeks in the isolated tube placement group and 6.5 weeks in the combined treatment group. In the overall cohort, the median preoperative VAS score was 6. In the subgroup analysis, the median preoperative VAS score was 5 in the subungual tube placement alone group and 6 in the combined treatment group. At 1 week after surgery, the median VAS score decreased to 1 in the subungual tube placement alone group and to 2 in the combined treatment group. The only observed postoperative complication was transient localized bleeding, which resolved spontaneously within 72 h. No recurrence was observed in any of the 19 treated nail margins during follow-up through 12 months. Patients reported satisfaction with both cosmetic and functional outcomes. Three key procedural refinements were introduced during technique development to improve procedural feasibility and standardization.

**Conclusion:**

This IDEAL Phase 1 study demonstrates the technical feasibility and short-term safety of subungual tube placement combined with selective nail-fold wedge resection for moderate-to-severe onychocryptosis-associated paronychia. Early outcomes were characterized by favorable healing and rapid pain relief, with no recurrence observed during the available follow-up period. Further investigation is required to determine the optimal catheter design and refine patient-selection criteria. Future research should proceed to an IDEAL Phase 2 (Exploration) study incorporating a larger cohort, longer follow-up, and continued refinement and standardization of the technique.

## Introduction

Paronychia is an inflammatory disorder of the periungual tissues that may be associated with secondary infection and is commonly precipitated by trauma or chronic mechanical irritation. Onychocryptosis is one of the most common underlying causes of chronic and recurrent paronychia (1). Previous studies have reported substantial recurrence after conservative treatment, and recurrence rates of up to 73% have been reported after simple nail avulsion in patients with onychocryptosis-associated paronychia. (2) Despite the availability of numerous treatment strategies, no universally accepted approach currently exists, and many emerging techniques have been introduced without systematic evaluation. The present study describes a modified nail-preserving technique and reports its early clinical application within an IDEAL Phase 1 (Idea) prospective case series. The primary objectives were to evaluate technical feasibility, procedural safety, procedural refinement, and preliminary clinical outcomes associated with subungual tube placement combined with selective nail-fold wedge resection.

The technique was developed based on the clinical observation that symptom improvement often follows relief of mechanical conflict between the nail plate and the adjacent nail fold. However, existing surgical approaches may not consistently maintain separation between these structures during subsequent nail growth. Accordingly, a nail-preserving strategy was developed to provide sustained mechanical guidance of the nail plate while minimizing disruption of native nail anatomy.

## Methods

This prospective, single-arm, single-center case series was designed as an IDEAL Phase 1 (Idea) study. The study protocol was approved by the Ethics Committee of the Affiliated Hospital of Chengdu University of Traditional Chinese Medicine on July 1, 2024 (Approval No. 2024KL-069), and was registered with the Chinese Clinical Trial Registry (www.chictr.org.cn; Registration No. ChiCTR2400089982; Trial ID: 239546). Consistent with IDEAL Phase 1 recommendations, the study aimed to evaluate technical feasibility, procedural safety, procedural refinement, and preliminary clinical outcomes to inform subsequent IDEAL Phase 2 investigation.

Between July 2024 and September 2024, consecutive patients with onychocryptosis-associated paronychia were screened for eligibility and enrolled if they met the following inclusion criteria: (1) Age between 12 and 60 years. (2) Clinical diagnosis of onychocryptosis-associated paronychia, classified as Stage II or Stage III (moderate-to-severe) according to the Heifetz or Martinez-Nova grading systems. (3) Symptom duration of at least 1 month. (4) Failure of prior conservative treatment or patient preference for surgical management. (5) Ability and willingness to undergo surgery and comply with follow-up assessments. Exclusion criteria included inability to provide informed consent, pregnancy or lactation, incomplete recovery from previous nail surgery within 3 months, coagulation disorders, immunological diseases, diabetes mellitus, active infections requiring systemic antibiotics, such as cellulitis or osteomyelitis, psychiatric disorders, toe or finger fractures, other severe systemic diseases or complications, and known allergy to lidocaine.

Following enrollment, the choice of surgical procedure was determined according to predefined clinical criteria related to the severity of nail-fold hypertrophy, fibrosis, and disease recurrence. Patients underwent nail-fold wedge resection in addition to subungual tube placement when one or more of the following criteria were met: (1) marked nail-fold hypertrophy, defined as a distance of ≥5 mm from the lateral edge of the nail plate to the outer margin of the nail fold or complete coverage of the nail plate by hypertrophic soft tissue; (2) fibrotic thickening of the nail fold, characterized by a firm, rubbery texture on palpation and a symptom duration exceeding 6 months; or (3) recurrence after two or more previous partial or total nail-avulsion procedures. All remaining patients underwent subungual tube placement alone. This stratified treatment strategy was adopted to individualize surgical management according to disease severity while preserving the minimally invasive nature of the procedure whenever possible.

The primary outcome measures included recurrence rate, healing time, Visual Analog Scale (VAS) pain scores, and postoperative patient satisfaction. Healing time and recurrence rates were assessed on a per-margin basis, with bilateral nail margins analyzed as independent anatomical units. Pain and satisfaction were assessed on a per-patient basis. Consequently, healing time and recurrence were evaluated in 19 treated nail margins, whereas pain and satisfaction were evaluated in 11 patients. Healing was defined as complete resolution of local erythema, exudate, and tenderness within the nail groove. For patients undergoing combined nail-fold wedge resection, complete epithelialization of the surgical wound was additionally required. Assessments were performed at 1, 2, 4, and 8 weeks postoperatively. Recurrence was defined as the reappearance of pain, erythema, swelling, purulent discharge, granulation tissue, or nail-edge embedding at the previously treated site after initial healing. Recurrence was evaluated at 8 weeks and at 3-, 6-, and 12-month follow-up visits. Pain intensity was assessed at rest preoperatively and at 1 and 4 weeks postoperatively using a 10-point Visual Analog Scale (VAS; 0 = no pain; 10 = worst imaginable pain). Patient satisfaction with cosmetic and functional outcomes was categorized as satisfactory, fair, or unsatisfactory at the 8-week follow-up visit.

### Preoperative preparation

Routine preoperative evaluation included complete blood count testing, coagulation studies, and screening for infectious diseases (hepatitis B, hepatitis C, HIV, syphilis). During the preoperative consultation, patients received detailed counseling regarding the planned anesthesia, surgical procedure, expected benefits, potential risks, and postoperative care requirements. Written informed consent, including authorization for anesthesia, was obtained from all participants or their legal guardians prior to the intervention. On the day of surgery, patients were instructed to clean the affected digit, wear non-restrictive footwear, and be accompanied by at least one family member to assist with postoperative care and transportation.

### Intraoperative procedures

Patients were positioned supine, and the affected toe was prepared and draped in a sterile fashion. Local anesthesia was achieved using a digital nerve block with 1% lidocaine. After confirmation of adequate anesthesia, a digital tourniquet was applied at the base of the toe to provide a bloodless operative field, and tourniquet time was monitored throughout the procedure.

In patients allocated to combined treatment, selective nail-fold wedge resection was performed before tube placement. A crescent-shaped wedge excision of the hypertrophic nail fold, including the overlying skin and subcutaneous tissue, was performed along the anterior margin of the affected nail fold. The incision was closed primarily using non-absorbable sutures. This maneuver facilitated downward traction of the periungual tissue and improved exposure of the embedded nail margin.

**Figure 1:**
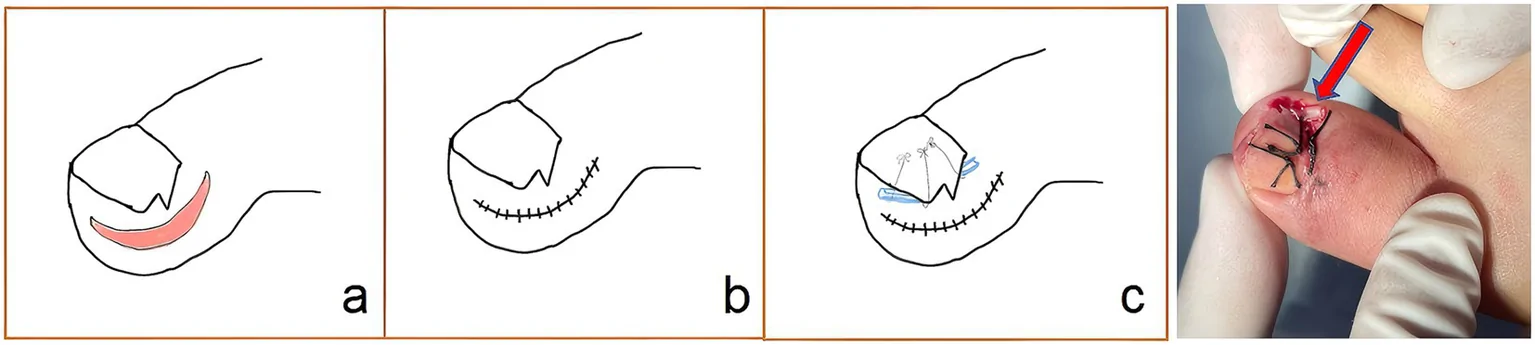
Surgical diagram for unilateral involvement. **(a)** Wedge resection is performed on the hypertrophic nail fold. **(b)** The wedge-shaped incision is sutured, and the periungual tissue at the lateral nail margin is pulled downward to expose the nail groove. **(c)** The trimmed plastic tube from a scalp vein catheter is inserted into the lateral nail groove between the lateral nail plate margin and the nail fold and secured with sutures. The red arrow indicates the subungual tube positioned longitudinally within the lateral nail groove between the lateral edge of the nail plate and the lateral nail fold.

**Figure 2:**
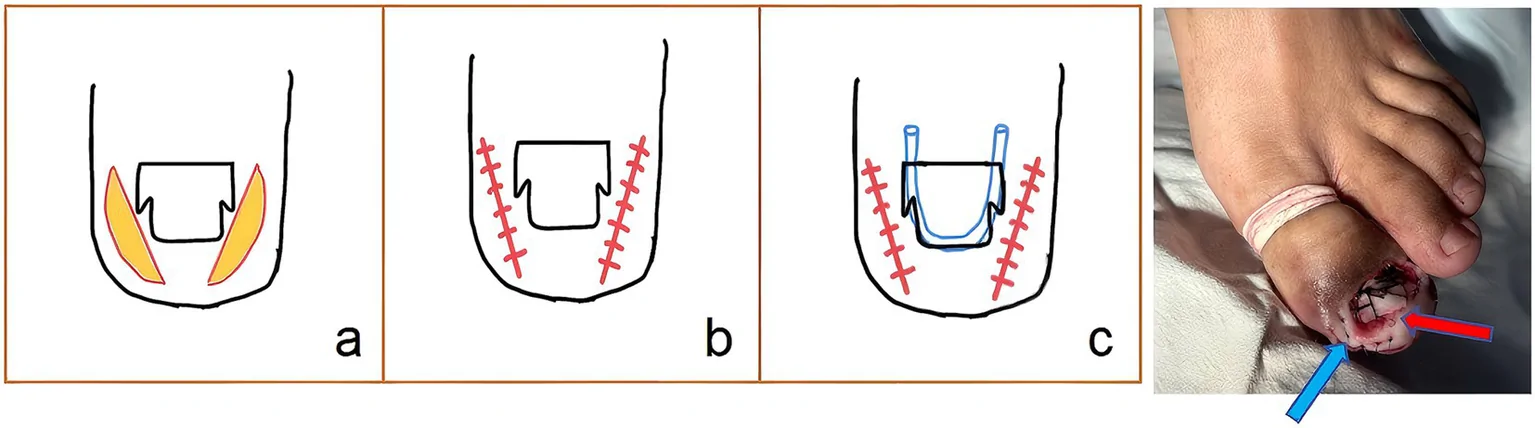
Surgical diagram for involvement of both nail margins. **(a)** Wedge resection is performed on the hypertrophic nail fold. **(b)** The wedge-shaped incision is sutured, and the periungual tissue at the affected nail margin is pulled downward to expose the nail groove. **(c)** The trimmed plastic tube from a scalp vein catheter is inserted into the nail groove between the nail plate margin and the nail fold and secured with sutures. The red arrow indicates the subungual tube positioned longitudinally within the affected nail groove between the nail plate margin and the nail fold. The blue arrow indicates the site of selective nail-fold wedge resection.

A scalp vein catheter (0.7 × 25 mm, TW LB) was trimmed to a length of 5–8 cm. Both ends were rounded using sterile scissors to minimize local tissue irritation, and the edges were checked under an operating microscope. The catheter was inserted into the lateral nail groove between the lateral nail plate margin and the nail fold. The catheter was positioned longitudinally within the lateral nail groove, with its proximal end 5 mm distal to the nail root and its distal end 1–2 cm beyond the free edge of the nail plate. The catheter was secured using 2–0 non-absorbable sutures to prevent displacement during nail growth, and any redundant material was trimmed. Following the procedure, the surgical site was cleansed and secured with a compression bandage, after which the hemostatic tourniquet was released. Patients remained in the observation suite for 30 min with the affected limb elevated to monitor for postoperative bleeding before discharge (Figures 1, 2).

### Postoperative dressing changes

Postoperative wound care included standardized dressing changes every 48 h. For patients who underwent the combined procedure, sutures from the wedge resection site were removed at 2 weeks postoperatively. The subungual catheter was maintained *in situ* for 8 weeks to provide sustained structural guidance for the growing nail plate and was then removed during a follow-up visit.

## Results

A total of 11 patients with onychocryptosis-associated paronychia were enrolled. Three patients underwent combined nail-fold wedge resection and subungual tube placement, whereas eight patients underwent subungual tube placement alone. The anatomical locations and corresponding surgical interventions for each patient are summarized in Table 1.

**Table 1:** Patient characteristics, anatomical involvement, and surgical procedures.

Case no.	1	2	3	4	5	6	7	8	9	10	11
Sex (F/M)	F	F	M	M	M	F	M	F	F	M	M
Age	25	25	15	30	30	17	18	16	16	19	13
Site	Bilateral halluces (medial and lateral margins)	Right hallux (lateral margins)	Left hallux (lateral margins)	Right hallux (medial side)	Right hallux (lateral margins)	Left hallux (lateral margins)	Bilateral halluces (medial and lateral margins)	Left hallux (lateral margins)	Left hallux (lateral margins)	Right hallux (medial and lateral margins)	Right hallux (medial and lateral margins)
Disease duration (y/mo)	10 y	2 y	6 mo	1 y	1 mo	1 y	1 y	1 mo	1 mo	6 mo	2 y
Recurrence status	No recurrence	No recurrence	No recurrence	No recurrence	No recurrence	No recurrence	No recurrence	No recurrence	No recurrence	No recurrence	No recurrence
Disease stage	III	II	II	II	II	II	III	III	III	III	II
Follow-up duration (mo)	17 mo	16 mo	14 mo	14 mo	14 mo	13 mo	13 mo	12 mo	13 mo	12 mo	12 mo
Outcome	Healed	Healed	Healed	Healed	Healed	Healed	Healed	Healed	Healed	Healed	Healed
Surgical procedure	Subungual tube placement combined with wedge resection	Subungual tube placement alone	Subungual tube placement alone	Subungual tube placement alone	Subungual tube placement alone	Subungual tube placement alone	Subungual tube placement combined with wedge resection	Subungual tube placement alone	Subungual tube placement alone	Subungual tube placement combined with wedge resection	Subungual tube placement alone

Participants ranged in age from 13 to 30 years, with a mean age of 20.4 years. The cohort included five female and six male patients. Regarding disease laterality, seven patients presented with unilateral onychocryptosis-associated paronychia, whereas four had bilateral involvement, yielding a total of 19 treated nail margins for analysis.

Clinical staging was based on the severity of onychocryptosis-associated paronychia and the Heifetz and Martinez-Nova classification systems, which were appropriately integrated and adapted for the study population (3–5) (Table 2), with six patients categorized as Stage II and 5 patients as Stage III. The mean disease duration from onset to surgical intervention was 19.9 months (range, 1–120 months). More than half of the patients had previously undergone nail avulsion. Notably, four patients had undergone nail avulsion more than once, and all had experienced postoperative recurrence before enrollment in this study.

**Table 2:** Severity grading of onychocryptosis-associated paronychia.

Disease stage	Symptoms
I	Pain in the lateral nail fold with erythema and swelling
II	Exacerbation of inflammation, local infection, and pus accumulation
III	Chronic inflammation with localized granulation tissue formation and hypertrophy of the nail fold

This classification was adapted from the Heifetz and Martinez-Nova staging systems for ingrown toenails. In the present study, Stage II was considered moderate disease, and Stage III was considered severe disease. Only patients with Stage II or Stage III disease were included.

All participants completed the scheduled postoperative follow-up assessments. The mean healing time was 5.8 weeks (range, 4–8 weeks) in patients treated with subungual tube placement alone and 6.5 weeks (range, 5–8 weeks) in those treated with combined subungual tube placement and nail-fold wedge resection. Preoperative pain levels were substantial. In the overall cohort, the median preoperative VAS score was 6. In subgroup analysis, the median preoperative VAS score was 5 in the subungual tube placement alone group and 6 in the combined treatment group. At 1 week after surgery, the median VAS score decreased to 1 in the subungual tube placement alone group and to 2 in the combined treatment group. At the 8-week postoperative assessment, all participants reported satisfactory cosmetic appearance and functional recovery. The median follow-up duration was 13 months (range, 12–17 months). All patients completed the 6- and 12-month follow-up assessments. No recurrence was observed in any of the 19 treated nail margins during follow-up. Given the exploratory nature of this IDEAL Phase 1 study and its limited sample size, formal statistical comparisons were not performed. Descriptive outcome data according to treatment group are provided in Table 3. Patient demographic characteristics, disease stage, and pain assessments were analyzed at the patient level (*n* = 11), whereas healing time and recurrence were analyzed at the treated nail-margin level (*n* = 19). Cases 1 and 7 had four margins each, Cases 10 and 11 had two margins each, and the remaining seven patients had one margin each, giving 19 treated nail margins in total. Follow-up beyond 12 months is ongoing to evaluate the long-term durability of the procedure and to monitor for late recurrence.

**Table 3:** Descriptive summary of patient-level and nail-margin-level outcomes.

Variable (patients/nail margins)	Overall (11/19)	Tube placement alone (8/9)	Combined treatment* (3/10)
Patient-level variables			
Age (Y), mean (range)	20.4 (13–30)	20.3 (13–30)	20.7 (18–25)
Sex (M/F), *n*	6/5	4/4	2/1
Disease severity stage (II/III), n	6/5	6/2	0/3
Disease duration (M), median (range)	12 (1–120)	6 (1–24)	12 (6–120)
Previous nail avulsion ≥1 time, *n* (%)	4 (36.4%)	1 (12.5%)	3 (100%)
Preoperative VAS score, median (range)	6 (5–7)	5 (5–6)	6 (6–7)
VAS score at 1 week postoperatively, median (range)	1 (1–3)	1 (1–2)	2 (2–2)
Follow-up duration (M), median (range)	13 (12–17)	13 (12–16)	13 (12–17)
Nail-margin-level variables			
Healing time (W), median (range)	6 (4–8)	5.5 (4–8)	7 (6–8)
Recurrence at final follow-up, *n* (%)	0 (0%)	0 (0%)	0 (0%)

Data were analyzed at two levels. Patient-level variables (*n* = 11) included age, sex, disease severity stage, disease duration, history of previous nail avulsion, preoperative VAS score, VAS score at 1 week, and follow-up duration. Treated nail-margin-level variables (*n* = 19) included healing time and recurrence at final follow-up. Cases 1 and 7 contributed four treated nail margins each; Cases 10 and 11 contributed two treated nail margins each; and each of the remaining seven patients contributed one treated nail margin, yielding a total of 19 treated nail margins.

## Discussion

### Surgical concept and design

Paronychia is an inflammatory condition of the periungual tissues of the fingernails or toenails and may be associated with secondary infection. It can be caused or aggravated by various factors, including trauma and chronic mechanical compression. Onychocryptosis is one of the most common underlying causes of recurrent paronychia (1). Current treatment options range from conservative management to a variety of surgical procedures, including partial or total nail avulsion, nail plate resection with or without matrixectomy (6), and the subungual thread method (7). Huang et al. (8) noted that the three major categories of surgical procedures for moderate-to-severe ingrown toenails each have distinct own advantages and disadvantages. Partial or total nail avulsion remains one of the most frequently performed procedures in primary care settings in China. Although it can rapidly relieve pain and remove the embedded nail edge, it does not necessarily correct the underlying mechanical conflict between the lateral nail plate and the nail fold. In addition, loss of mechanical pressure from the nail plate may lead to hyperplasia and thickening of the nail bed. This may subsequently cause the newly formed nail plate to penetrate the distal nail bed, resulting in distal ingrown toenail. Matrixectomy may reduce recurrence by permanently narrowing the nail plate; however, it is inherently destructive and may compromise cosmetic appearance. Wedge resection or nail-fold excision may reduce the volume of hypertrophic periungual tissue; however, when performed alone, these procedures may not provide sustained guidance for the regrowing nail plate.

The conceptual basis of the present technique is the hypothesis that persistent mechanical compression between the lateral nail plate and the nail fold contributes to persistent symptoms and recurrence. Accordingly, maintaining separation during nail growth may help restore a more normal relationship between the nail plate and the nail fold. Therefore, the objective of the technique is to provide temporary mechanical guidance while preserving the native nail structure.

Previously described splinting techniques, including gutter splinting, groove splinting, and flexible tube splinting, are based on a similar principle of mechanical separation. Accordingly, the use of a tube spacer itself should not be considered novel. Rather, the distinguishing features of the present approach include prolonged indwelling tube guidance, suture-based fixation, selective combination with nail-fold wedge resection, and stratified treatment selection based on disease severity and recurrence history. These modifications were introduced through iterative procedural refinement and evaluated within the IDEAL framework.

Compared with previously reported tube-splinting techniques, the present approach was developed to address three practical limitations encountered during routine clinical practice. First, temporary or inadequately secured spacers may become displaced before the lateral nail margin grows beyond the nail groove. In the present study, the catheter was fixed with non-absorbable sutures and retained for approximately 8 weeks to provide prolonged mechanical guidance. Second, in patients with marked nail-fold hypertrophy or fibrotic hyperplasia, tube placement alone may not provide adequate decompression because the hypertrophic fold can compress the tube and perpetuate mechanical conflict between the nail plate and the nail fold. For this subgroup, selective nail-fold wedge resection was combined with tube placement to decompress the nail groove. Third, a stratified treatment algorithm may reduce unnecessary tissue resection in milder cases while providing additional decompression in severe cases.

### Technique evolution during the IDEAL Phase 1 study

#### Stratified selection of the surgical procedure

During the ethically approved IDEAL Phase 1 study, the operative concept remained consistent, but several technical details were prospectively refined based on intraoperative and postoperative observations. Early clinical experience suggested that (9) isolated tube placement could be insufficient in patients with marked fibrotic nail-fold hypertrophy because persistent soft-tissue compression limited effective decompression of the nail groove (10, 11). Alkhalifah et al. (12) emphasized the importance of tailoring treatment selection according to disease severity and prior treatment history. Consistent with this principle, treatment selection in the present study was stratified based on the severity of nail-fold hypertrophy and the patient’s treatment history. To reduce inter-operator variability and maintain procedural consistency during technique development, all procedures were performed by a single experienced surgeon.

#### Optimization of the puncture site for tube placement

Stable fixation and appropriate positioning of the subungual catheter were considered important for maintaining catheter stability throughout the treatment period. Clinical observations suggested that the catheter fixation pathway was most suitable when aligned with the lateral nail margin. The exit point was subsequently relocated to approximately 2–3 mm distal to the proximal nail fold. This orientation bypasses the highly vascularized region of the proximal nail fold, thereby minimizing mechanical trauma to the nail bed and reducing the risk of postoperative bleeding.

#### Standardization of catheter indwelling duration

Clinical experience also suggested that catheter retention should continue until the lateral nail margin had grown beyond the nail groove, which typically required approximately 8 weeks in the present cohort. This duration appeared sufficient to maintain separation during nail growth and may reduce the likelihood of recurrent embedding.

These observations were prospectively documented in the ethically approved 11-patient cohort and informed the procedural refinements reported in this IDEAL Phase 1 study. The three preliminary cases treated in routine clinical practice before formal study enrollment were not included in the present cohort and were not analyzed as study outcomes. These cases only helped formulate the initial operative concept before the start of the IDEAL Phase 1 study. All reported outcomes and IDEAL Phase 1 refinements were derived from prospectively documented intraoperative and postoperative observations in the ethically approved 11-patient cohort.

### Study conclusions

Recurrence remains a major challenge in the management of onychocryptosis-associated paronychia, and currently available surgical procedures vary considerably in recurrence rates, cosmetic outcomes, and degree of tissue preservation. The present technique combines prolonged tube-guided nail growth with selective nail-fold wedge resection in patients with marked hypertrophy and fibrosis, aiming to reduce mechanical compression and guide nail growth while preserving the native nail plate. Kocak and Ozyalcin (13) reported a recurrence rate of 14% after the modified Winograd procedure, whereas groove-splinting techniques have been described as nail-preserving alternatives for the management of ingrown nails (14, 15). Although the present technique shares conceptual similarities with previously reported splinting methods, prolonged tube retention, suture-based fixation, and stratified treatment selection represent important procedural modifications. The absence of observed recurrence in the present cohort is encouraging; however, the small sample size, non-comparative design, and limited follow-up duration preclude conclusions regarding recurrence reduction or superiority over existing treatments.

The favorable healing outcomes and rapid pain reduction observed in this study suggest that the technique is technically feasible and warrants further investigation. Nevertheless, larger comparative studies are required to determine long-term durability, patient-reported outcomes, and relative effectiveness compared with established surgical approaches (13, 16).

### Future prospects

The current technique uses a polyethylene catheter derived from a scalp vein set, which requires manual intraoperative customization based on the surgeon’s judgment. This manual process may introduce technical variability. An excessively large catheter diameter may elevate the nail plate excessively, potentially resulting in secondary onycholysis. Conversely, a catheter with an insufficient diameter may fail to achieve adequate separation between the nail plate and the surrounding soft tissue, potentially leading to persistent onychocryptosis. Furthermore, surface irregularities along the trimmed catheter margins may cause chronic friction-related irritation during prolonged indwelling. Future development should focus on specialized, prefabricated spacers with standardized dimensions to improve procedural efficiency, enhance reproducibility, and reduce patient discomfort.

This study included only 11 patients and was limited by a small sample size, a narrow age range, and a relatively short observation period. Therefore, the present findings support only the technical feasibility, preliminary safety, and short-term clinical improvement of the technique. They cannot establish superiority over nail avulsion, matrixectomy, wedge resection, or previously reported splinting techniques. Larger IDEAL Phase 2 studies and subsequent comparative trials are required to evaluate recurrence, long-term durability, patient-reported outcomes, and relative effectiveness. Future studies should enroll larger cohorts to allow more comprehensive observation and further refinement of the surgical protocol. Furthermore, in susceptible populations, particularly patients with diabetes, paronychia may contribute to foot-related complications (17–19). Because this was an initial feasibility study of a modified technique, patients with diabetes were not enrolled in order to minimize confounding in the safety assessment. Therefore, the results cannot be directly extrapolated to patients with diabetic foot disease. Future studies involving patients with diabetes are needed to further evaluate the safety and effectiveness of this technique in this population. In addition, the duration of subungual catheter retention was fixed at 8 weeks in the present study. However, whether the optimal duration varies according to patient age or nail growth rate remains unclear. The choice between subungual tube placement alone and combined treatment was based primarily on clinical judgment and lacked precise quantitative criteria. Patient satisfaction with cosmetic and functional outcomes was not assessed using a validated scale in the present study. Future studies should incorporate validated and standardized outcome measures to provide more objective and reliable assessments.

Based on these preliminary findings, the next step should be an IDEAL Phase 2 exploratory study. Future studies should enroll a larger cohort, perform more comprehensive follow-up assessments, and further refine the surgical protocol. Specific measures will include expanding the sample size to approximately 50 patients through continuous enrollment, developing a standardized protocol for catheter preparation, exploring quantitative criteria for patient selection, and establishing a standardized outcome-reporting system for future comparative studies.

This IDEAL Phase 1 study supports the technical feasibility and short-term safety of subungual tube placement, either alone or combined with selective nail-fold wedge resection, for moderate-to-severe onychocryptosis-associated paronychia. Early outcomes were characterized by favorable healing, rapid pain relief, and no recurrence during the available follow-up period. Remaining uncertainties regarding catheter design, patient selection, and procedural standardization require further evaluation in subsequent IDEAL Phase 2 studies. This study provides preliminary evidence supporting further investigation of this modified nail-preserving technique.

## Data Availability

The raw data supporting the conclusions of this article will be made available by the authors, without undue reservation.

## References

[1] Olamiju B Bhullar S Coleman EL Leventhal JS. Management of Paronychia with Pseudopyogenic granulomas secondary to epidermal growth factor receptor inhibitors: an assessment of topical Timolol and the need for multiple medical and procedural therapies. J Am Acad Dermatol. (2021) 84:806–8. doi: 10.1016/j.jaad.2020.06.010, 32526318

[2] Grieg JD Anderson JH Ireland AJ Anderson JR. The surgical treatment of ingrowing toenails. J Bone Joint Surg Br. (1991) 73:131–3. doi: 10.1302/0301-620x.73b1.1991748, 1991748

[3] Martínez-Nova A Sánchez-Rodríguez R Alonso-Peña D. A new Onychocryptosis classification and treatment plan. J Am Podiatr Med Assoc. (2007) 97:389–93. doi: 10.7547/0970389, 17901344

[4] Heifetz CJ. Ingrown toe-nail: a clinical study. Am J Surg. (1937) 38:298–315.

[5] Zaraa I Dorbani I Hawilo A Mokni M Ben Osman A. Segmental phenolization for the treatment of ingrown toenails: technique report, follow up of 146 patients, and review of the literature. Dermatol Online J. (2013) 19:18560. doi: 10.5070/D3196018560, 24011310

[6] Kayalar M Bal E Toros T Ozaksar K Gürbüz Y Ademoğlu Y. Results of partial Matrixectomy for chronic ingrown toenail. Foot Ankle Int. (2011) 32:888–95. doi: 10.3113/fai.2011.0888, 22097165

[7] Ince B Dadaci M Altuntas Z. Knot technique: a new treatment of ingrown nails. Dermatol Surg. (2015) 41:250–4. doi: 10.1097/dss.0000000000000271, 25627634

[8] Huang S Wang J Chen Z Kang Y. Surgical interventions for ingrown toenail. Foot Ankle Surg. (2024) 30:181–90. doi: 10.1016/j.fas.2023.12.003, 38177051

[9] Persichetti P Simone P Li Vecchi G Di Lella F Cagli B Marangi GF. Wedge excision of the nail fold in the treatment of ingrown toenail. Ann Plast Surg. (2004) 52:617–20. doi: 10.1097/01.sap.0000095436.08812.67, 15167000

[10] Gupta S Sahoo B Kumar B. Treating ingrown toenails by nail splinting with a flexible tube: an Indian experience. J Dermatol. (2001) 28:485–9. doi: 10.1111/j.1346-8138.2001.tb00016.x, 11603389

[11] Nazari S. A simple and practical method in treatment of ingrown nails: splinting by flexible tube. J Eur Acad Dermatol Venereol. (2006) 20:1302–6. doi: 10.1111/j.1468-3083.2006.01793.x, 17062049

[12] Alkhalifah A Dehavay F Richert B. Management of ingrowing nail. Hand Surg Rehabil. (2024) 43S:101628. doi: 10.1016/j.hansur.2023.12.002 38128646

[13] Kocak S Ozyalcin A. Modified Winograd technique versus partial resection with electrocoagulation for surgical management of ingrown toenails. J Orthop Surg Res. (2025) 20:361. doi: 10.1186/s13018-025-05677-4, 40205468 PMC11983720

[14] Erdem O Dağtaş BB Koku Aksu AE Göktay F. Suture-secured modified gutter splint method for the conservative treatment of ingrown toenails. J Am Acad Dermatol. (2023) 88:e301. doi: 10.1016/j.jaad.2021.02.021, 33582261

[15] Ceren E Gokdemir G Arikan Y Purisa S. Comparison of phenol Matricectomy and nail-splinting with a flexible tube for the treatment of ingrown toenails. Dermatol Surg. (2013) 39:1264–9. doi: 10.1111/dsu.12230, 23631560

[16] Exley V Jones K O'Carroll G Watson J Backhouse M. A systematic review and meta-analysis of randomised controlled trials on surgical treatments for ingrown toenails part I: recurrence and relief of symptoms. J Foot Ankle Res. (2023) 16:35. doi: 10.1186/s13047-023-00631-1, 37301845 PMC10257290

[17] Chuback J Embil JM Sellers E Trepman E Cheang M Dean H. Foot abnormalities in Canadian aboriginal adolescents with type 2 diabetes. Diabet Med. (2007) 24:747–52. doi: 10.1111/j.1464-5491.2007.02133.x, 17403123

[18] Vural S Bostanci S Koçyigit P Çaliskan D Baskal N Aydin N. Risk factors and frequency of ingrown nails in adult diabetic patients. J Foot Ankle Res. (2018) 57:289–95. doi: 10.1053/j.jfas.2017.10.006, 29329712

[19] Azuma H Ikura K Miura J Babazono T. A fact-finding survey on pre-ulcerative lesions of foot in patients with diabetes: analysis using the diabetes study from the Center of Tokyo Women's medical university 2018 (DIACET 2018). Diabetol Int. (2023) 14:397–405. doi: 10.1007/s13340-023-00649-7, 37781473 PMC10533771

