# Supplementary material for: IDEAL Phase 1 evaluation of subungual tube placement with selective nail-fold wedge resection for moderate-to-severe onychocryptosis-associated paronychia
Source: Front Med (Lausanne). 2026 Aug 12;13:1822211. doi: 10.3389/fmed.2026.1822211 (PMC13506480; doi:10.3389/fmed.2026.1822211)
Supplement: Supplementary file 1 [file Supplementary_file_1.DOCX]

**Supplementary**

**Case Presentation**

Case 1 to Case 11(Fig. 5)

Case 1: Female, 25 years old, stage III, bilateral involvement of both great toes (10-year disease duration, multiple prior nail avulsions). Underwent Wedge Resection of the Nail Fold Combined with Subungual Tube Placement
1a: Preoperative: Marked hypertrophy of the bilateral nail folds, erythema of the nail folds, and severe ingrown toenail.
1b: Immediately postoperatively: Bilateral subungual catheters secured in place; wedge incisions well-sutured; no active bleeding.
1c: 1 week postoperatively: Incisions dry without erythema; catheters in place; patient reported no pain.
1d: 2 weeks postoperatively: Nail fold morphology restored; nail plate beginning to grow outward and upward.

Case 2: Female, 25 years old, stage II, lateral side of the right great toe. Underwent Subungual Tube Placement
2a: Preoperative: Erythema of the lateral nail fold of the right great toe with ingrown nail embedded.
2b: Immediately postoperatively: Catheter inserted between the nail plate and nail bed and secured with suture.
2c: 1 week postoperatively: Catheter in place; nail fold erythema resolved; no wound exudate.
2d: 2 weeks postoperatively: Nail plate growing well; catheter retained; no discomfort.
2e: 1 month postoperatively: Nail plate growing well; catheter retained; no discomfort.
2f: 2 months postoperatively: Catheter removed; nail plate growing outward and upward; nail fold morphology normal; no recurrence.

Case 3: Male, 15 years old, stage II, lateral side of the left great toe. Underwent Subungual Tube Placement
3a: Preoperative: Erythema of the lateral nail fold of the left great toe with obvious ingrown nail.
3b: 1 day postoperatively: Catheter in place; no wound bleeding.
3c: 1 week postoperatively: Catheter well-secured; nail fold erythema largely resolved.
3d: 2 weeks postoperatively: Catheter well-secured; nail plate growing outward and upward.
3e: 2 months postoperatively: Mild bleeding after catheter removal, which ceased after 5 minutes of local compression; no signs of infection.

Case 4: Male, 30 years old, stage II, medial side of the right great toe. Underwent Subungual Tube Placement
4a: Preoperative: Erythema of the medial nail fold of the right great toe with embedded ingrown nail.
4b: Immediately postoperatively: Catheter inserted and secured.
4c: 1 week postoperatively: Catheter in place; nail fold erythema markedly reduced.
4d: 2 weeks postoperatively: Catheter well-secured; nail plate growing outward and upward.
4e: 1 month postoperatively: Nail plate growing well; catheter in place.
4f: 2 months postoperatively: Catheter removed; nail plate growing outward and upward; no erythema of the nail fold.

Case 5: Male, 30 years old, stage II, lateral side of the right great toe. Underwent Subungual Tube Placement

5a: Preoperative: The lateral nail fold of the right great toe with obvious ingrown nail.
5b: Immediately postoperatively: Catheter inserted and secured.
5c: 1 week postoperatively: Catheter in place; nail fold erythema largely resolved.
5d: 2 weeks postoperatively: Catheter well-secured; nail plate growing outward and upward.
5e: 1 month postoperatively: Nail plate growing well; catheter in place.
5f: 2 months postoperatively: Catheter removed; nail plate growing outward and upward; satisfactory cosmetic outcome.

Case 6: Female, 17 years old, stage II, lateral side of the left great toe. Underwent Subungual Tube Placement
6a: Preoperative: The lateral nail fold of the left great toe with embedded ingrown nail.
6b: Immediately postoperatively: Catheter inserted and secured.
6c: 2 weeks postoperatively: Catheter in place; nail fold erythema largely resolved.
6d: 1 month postoperatively: Nail plate growing well; catheter in place.
6e: 2 months postoperatively: Catheter removed; nail plate growing outward and upward; normal nail fold morphology.

Case 7: Male, 18 years old, stage III, bilateral involvement of both great toes (1-year disease duration). Underwent Wedge Resection of the Nail Fold Combined with Subungual Tube Placement
7a: Preoperative: Hypertrophy of bilateral nail folds; nail fold erythema with granulation tissue formation (notable on the right side).
7b: Immediately postoperatively: Bilateral subungual catheters secured; wedge incisions well-sutured.
7c: 1 week postoperatively: Mild granulation tissue formation on the medial side of the right great toe, managed during dressing change.
7d: 2 weeks postoperatively: Wedge incision sutures removed; nail fold morphology restored.

7e: 1 month postoperatively: Nail plate growing well; catheters in place.
7f: 2 months postoperatively: Catheters removed; bilateral nail plates growing outward and upward; no recurrence.

Case 8: Female, 16 years old, stage III, lateral side of the left great toe. Underwent Subungual Tube Placement
8a: Preoperative: Hypertrophy of the lateral nail fold of the left great toe; nail fold erythema with embedded ingrown nail.
8b: Immediately postoperatively: Catheter inserted and secured.
8c: 1 week postoperatively: Catheter in place; nail fold erythema markedly reduced.
8d: 2 weeks postoperatively: Nail plate growing well; catheter in place.
8e: 2 months postoperatively: Nail plate growing outward and upward; no erythema of the nail fold; satisfactory cosmetic outcome.

Case 9: Female, 16 years old, stage III, lateral side of the left great toe. Underwent Subungual Tube Placement
9a: Preoperative: Erythema of the lateral nail fold of the left great toe; nail fold hypertrophy with obvious ingrown nail.
9b: Immediately postoperatively: Catheter inserted and secured.
9c: 1 week postoperatively: Catheter in place; nail fold erythema largely resolved.
9d: 2 months postoperatively: Nail plate growing outward and upward; no erythema of the nail fold; satisfactory cosmetic outcome.

Case 10: Male, 19 years old, stage III, bilateral involvement of the right great toe. Underwent Wedge Resection of the Nail Fold Combined with Subungual Tube Placement

10a: Preoperative: Hypertrophy of bilateral nail folds of the right great toe; nail fold erythema with embedded ingrown nail.
10b: 1 week postoperatively: Bilateral catheters in place; wedge incisions healing well; nail fold erythema resolved.
10c: 2 weeks postoperatively: Sutures removed; wound healed well; Catheter in place.
10d: 2 months postoperatively: Nail plates growing outward and upward; bilateral nail fold morphology normal; no recurrence.

Case 11: Male, 13 years old, stage II, bilateral involvement of the right great toe (pincer nail deformity). Underwent Subungual Tube Placement
11a: Preoperative: Pincer nail deformity of the right great toe with bilateral ingrown toenail embedding.
11b: Preoperative: Marked curvature of the nail plate embedding into the nail folds.
11c: Immediately postoperatively: Bilateral catheters secured.
11d: 2 weeks postoperatively: Nail plate partially flattened and grew outward and upward; nail folds without erythema.
